# N-Acetylcysteine-Functionalized Mixed Micelles Overcome Multiple Intestinal Barriers to Improve Oral Bioavailability and Antioxidant Protection of Imperatorin

**DOI:** 10.3390/pharmaceutics18081036

**Published:** 2026-08-20

**Authors:** Yu Zhang, Jian Guo, Haonan Qiu, Jiale Liu, Chi Zhang, Lutan Zhou, Chunfei Wang, Lihua Li, Xuefeng Hou

**Affiliations:** 1 Drug Research and Development Center, School of Pharmacy, Wannan Medical University, Wuhu 241002, China; 20249171@stu.wnmc.edu.cn (Y.Z.); 20249157@stu.wnmc.edu.cn (H.Q.); 20259198@stu.wnmc.edu.cn (J.L.); 22107070024@stu.wnmc.edu.cn (C.Z.); 20210007@wnmc.edu.cn (L.Z.); 20220028@wnmc.edu.cn (C.W.); 2 Anhui Province Key Laboratory of Pharmaceutical Preparation Technology and Application, Anhui University of Chinese Medicine, Hefei 230012, China; guojian@ahtcm.edu.cn; 3 Anhui Provincial Engineering Laboratory for Screening and Re-Evaluation of Active Compounds of Herbal Medicines in Southern Anhui, Wannan Medical University, Wuhu 241002, China; 4 Anhui Provincial Engineering Research Center for Polysaccharide Drugs, Wannan Medical University, Wuhu 241002, China; 5 Anhui Innovative Center for Drug Basic Research of Metabolic Diseases, Wannan Medical University, Wuhu 241002, China

**Keywords:** imperatorin, N-acetylcysteine, mixed micelles, oral delivery, mucus penetration, bioavailability, antioxidant protection

## Abstract

**Background**: Imperatorin (IPT) is a natural furanocoumarin featuring robust anti-inflammatory, antifibrotic and antioxidant activities. However, poor aqueous solubility and insufficient oral bioavailability restrict its clinical application. Multiple gastrointestinal barriers, including the mucus barrier, limited epithelial penetration and P-glycoprotein-triggered drug efflux, are major obstacles hindering IPT oral absorption. **Methods**: N-acetylcysteine (NAC)-functionalized TPGS conjugates were synthesized first. Using Pluronic^®^ F108 and Lipoid^®^ S-100 as a matrix, imperatorin@N-acetylcysteine-TPGS/Pluronic^®^ F108/Lipoid^®^ S-100 (IPT@NAC-TFS) micelles were fabricated. We characterized their physicochemical features and in vitro release behavior. The Caco-2/HT29-MTX-E12 co-culture cell model was adopted to explore mucus permeation, cellular uptake and transepithelial transport mechanisms. In vivo intestinal distribution and pharmacokinetic tests in rats were carried out to confirm the oral absorption-enhancing effect of micelles. **Results**: Optimized micelles displayed a uniform shape and favorable encapsulation efficiency. Low CMC maintained structural stability upon gastrointestinal dilution. NAC modification conferred mucus-penetrating capacity on micelles. TPGS simultaneously improved epithelial barrier permeability and inhibited drug efflux, switching IPT transport mode. The micelles effectively cleared intracellular ROS, recovered SOD activity and lowered MDA levels in BLM-impaired MLg fibroblasts. In vivo results revealed enhanced intestinal drug accumulation, with the relative oral bioavailability of IPT increased by 6.07-fold. **Conclusions**: IPT@NAC-TFS micelles overcome multiple gastrointestinal barriers for oral IPT delivery. Combining mucus penetration, efflux suppression and antioxidative capacity, this system offers a promising strategy to develop oral formulations of poorly soluble antifibrotic natural products.

## 1. Introduction

Imperatorin (IPT), as the main extract from the traditional Chinese medicine *Angelica dahurica*, is a furocoumarin compound [1]. Modern pharmacological studies have shown that IPT has a wide range of physiological activities and high medicinal value. It not only has anti-inflammatory and antioxidant effects [2,3] but also possesses good antifibrotic properties [4,5], which suggests that it may have potential application prospects in fibrotic diseases. However, as a lipophilic molecule, IPT exhibits poor solubility in aqueous media [6]. This inherent property hinders the dissolution of IPT in the gastrointestinal lumen, ultimately leading to insufficient oral absorption and narrow clinical applicability [7]. In this context, it is imperative to develop effective strategies to improve the oral bioavailability of IPT for further translational application.

Oral delivery remains the most prevalent and patient-friendly administration strategy for clinical medication [8,9]. However, orally ingested drugs need to traverse a series of physiological obstacles before entering the systemic circulation. These agents first withstand the harsh acidic and digestive environment in the stomach, penetrate the mucus blanket covering the intestinal epithelial surface, and, finally, cross the tight intestinal epithelial barrier to achieve absorption [10,11,12]. Even if drugs successfully enter intestinal epithelial cells through transcellular pathways, they still face excretion resistance from efflux transporters. These membrane proteins pump intracellular drug substrates back into the intestinal cavity, greatly reducing the overall absorption efficiency of oral IPT [13,14]. The mucus barrier is composed of the mucin network secreted by the gastrointestinal epithelium, and it contains a dense and porous network structure that can physically retain large-sized particles [15]. At the same time, it relies on hydrophobic and electrostatic interactions to hinder the transport of hydrophobic small molecules [16]. If the drug fails to rapidly penetrate the mucus layer within 3–4 h, it will be affected by intestinal emptying and difficult to be absorbed. Therefore, it is necessary for the drug to rapidly penetrate the mucus layer [17]. This requires controlling its particle size within 200 nm and having a hydrophilic surface [18]. The hydrophilic surface can not only reduce the interaction with mucin through spatial steric hindrance but also inhibit non-specific protein adsorption and particle aggregation, improving mucosal biocompatibility and enhancing mucosal penetration ability [19]. In addition, electrically neutral drugs can maximize the overcoming of electrostatic adsorption, thereby improving the efficiency of transmucosal absorption [20]. Although lipophilic drugs readily traverse the small intestinal epithelial barrier, they are frequently recognized by efflux proteins such as P-gp located on the apical membrane of epithelial cells and subsequently pumped back into the intestinal lumen, which markedly impairs drug absorption efficiency [21]. Nevertheless, this lipophilic property is, to a certain extent, incompatible with the hydrophilic characteristics required for penetrating the mucus layer, which constitutes a major obstacle to efficient oral drug delivery. Accordingly, novel drug delivery systems capable of overcoming multiple physiological barriers during oral absorption, including gastrointestinal physicochemical barriers, mucus barriers, small intestinal epithelial barriers and efflux transporter barriers, are expected to become a core strategy for improving the oral bioavailability of IPT and achieving its maximal pharmacological efficacy.

In recent years, the mixed micellar nano-delivery system has demonstrated outstanding application potential in improving the dissolution behavior of poorly soluble drugs, enhancing stability in both in vivo and in vitro conditions and achieving targeted delivery and controlled release regulation. Compared with standard micelles, the critical micelle concentration (CMC) of mixed micelles is lower, their structure is more stable, and they have a higher drug loading capacity and encapsulation rate for hydrophobic drugs. The hydrophobic core constructed by multiple components also provides an ideal storage environment for poorly soluble drugs, further enhancing the application potential of this type of drug delivery system for oral administration [22]. The mixed micelles are formed by the co-assembly of multiple amphiphilic components and can be flexibly adjusted to regulate the overall hydrophilic-hydrophobic balance of the micelles through precise control of the ratio of different amphiphilic polymers [23]. The hydrophobic core can efficiently encapsulate lipophilic active molecules, and, at the same time, the thickness of the hydrophilic shell and the distribution of nanoparticle size can be regulated [24]. On this basis, the delivery performance can be precisely manipulated to address the limitations of poorly soluble drugs, including low aqueous solubility and a tendency toward precipitation. Furthermore, mixed micelles possess favorable structural plasticity and are highly amenable to modification with targeting ligands. The self-assembled micelles exhibit a typical core–shell architecture. The hydrophilic outer layer of micelles stretches into the surrounding aqueous gastrointestinal environment, and abundant functional groups on the micelles’ surface provide sufficient reactive sites for the immobilization of targeted ligands [25,26]. Moreover, surface-modified targeting moieties on mixed micelles are capable of specifically binding to highly expressed receptors on intestinal epithelial cells, which further initiates the cellular endocytosis process [27]. Unlike small-molecule drugs that enter cells merely through passive transmembrane diffusion, the endocytic uptake mechanism bypasses the recognition and excretion effects of membrane-resident P-gp and other efflux pumps. This unique characteristic markedly elevates intracellular drug accumulation in intestinal epithelial cells, facilitates the basolateral transmembrane transport of drugs into systemic blood circulation, and eventually achieves a substantial improvement in the intestinal absorption of poorly soluble therapeutic agents [28].

Despite the numerous advantages of mixed micelles, effective mucus penetration and subsequent internalization by small intestinal epithelial cells are highly dependent on elaborate structural design and surface functional modification. NAC is a safe, highly effective mucolytic compound with good water solubility, and it has been clinically used to relieve respiratory diseases characterized by dense, hard-to-clear mucus. Free thiol groups in NAC molecules break disulfide linkages among mucin polypeptide chains. Such cleavage reduces mucus viscosity and elasticity and destroys the cross-linked mesh architecture of mucus [29]. Immobilizing NAC on micellar surfaces facilitates the penetration of mixed micelles through viscous mucus layers to access intestinal epithelial surfaces. Apart from mucolytic effects, NAC serves as a powerful antioxidant that eliminates reactive oxygen species in the gastrointestinal lumen and intracellular compartments [30]. It alleviates carrier degradation and drug oxidative injury triggered by oxidative stress so as to maintain drug stability throughout mucosal transport.

Vitamin E polyethylene glycol succinate (D-α-Tocopherol polyethylene glycol 1000 succinate, TPGS) is an emerging nonionic surfactant. Consisting of lipophilic vitamin E moieties and hydrophilic long polyethylene glycol chains, this molecule exhibits typical amphipathic properties [31,32]. Incorporated into the outer shell of mixed micelles, TPGS strengthens the interaction between mixed micelles and intestinal epithelial cell membranes, thereby facilitating membrane fusion and cellular uptake. Moreover, TPGS is able to hinder the ATPase activity of membrane-bound P-gp in small intestinal epithelial cells and block P-gp-driven IPT efflux. Such effects extend the intracellular residence time of IPT, boost its transcellular transportation, and, accordingly, achieve prominent enhancement of the oral bioavailability of this compound [33].

Given that the performance of mixed micelles is highly dependent on the rational selection of amphiphilic building blocks, Pluronic^®^ F108, a polyethylene oxide–polypropylene oxide block copolymer, was selected as the primary matrix. Pluronic^®^ F108 exhibits excellent self-assembly capacity and can participate in constructing the hydrophobic core of mixed micelles to encapsulate lipophilic IPT. Meanwhile, its molecular structure contains a high proportion of hydrophilic polyethylene oxide (PEO) segments, which can form a hydration layer on the surface of the micelles, weakening the hydrophobic and electrostatic interactions between the micelles and the gastrointestinal mucin and endowing the micelles with good mucin penetration ability [34]. Lipoid^®^ S-100 can promote the fusion of the mixed micelles with the intestinal epithelial cell membrane, assist in activating the endocytosis pathway, and enhance drug transcellular transport, and the phospholipid structure can strengthen the hydrophobic core of the micelles [35], increasing the drug loading capacity for lipophilic IPT.

**Scheme 1:**
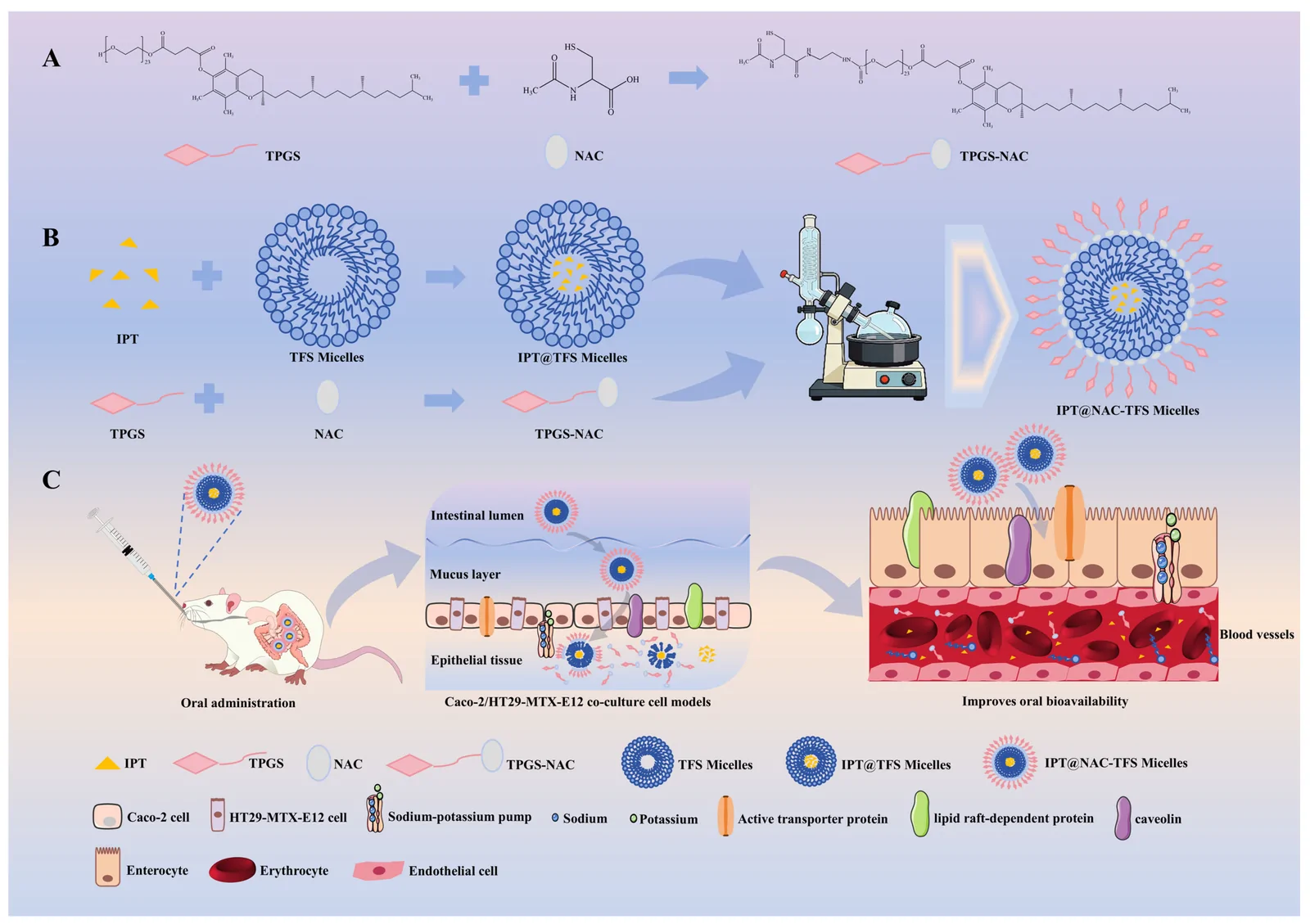
(**A**) The synthetic route of TPGS-NAC. (**B**) Schematic diagram of the preparation process of IPT@NAC-TFS micelles. (**C**) Schematic diagram of IPT@NAC-TFS micelles penetrating the mucus barrier of Caco-2/HT29-MTX-E12 cells and enhancing the bioavailability of IPT in rats after oral administration.

Aiming to overcome the multiple gastrointestinal physiological barriers that limit the oral absorption and clinical translation of IPT, this study rationally designed a multifunctional mixed micelles delivery system to address the poor water solubility and low oral bioavailability of lipophilic IPT (Scheme 1). Briefly, a dual-functional TPGS-NAC conjugate was synthesized and structurally validated. IPT@NAC-TFS mixed micelles were fabricated using Pluronic^®^ F108 and Lipoid^®^ S-100 as carrier matrices, and their basic physicochemical properties and in vitro release characteristics under simulated intestinal conditions were systematically characterized. This study adopted a Caco-2/HT29-MTX-E12 co-culture cell model to assess the capacity of mixed micelles to cross the dual mucus–epithelial intestinal barrier. Relevant evaluation indicators covered cellular uptake behavior, internalization pathways and transmembrane transport efficiency. In vitro oxidative stress experiments were also performed to validate the protective antioxidant effects of optimized micelles on damaged fibroblasts under oxidative injury. Consistent with in vitro findings, in vivo pharmacokinetic tests confirmed that the fabricated micelles remarkably boosted the oral bioavailability of IPT. Overall, the constructed multifunctional micellar system remedies the inherent delivery defects of raw IPT. It comprehensively improves drug solubility, mucosal penetrating ability, cellular internalization efficiency and antioxidant performance, thereby overcoming the bottlenecks restricting the oral delivery of IPT. This work provides reliable experimental and theoretical support for the development of high-bioavailability IPT formulations and broadens the application prospects.

## 2. Materials and Methods

### 2.1. Materials

IPT (≥98% purity) was supplied by Nanjing Casses Pharmaceutical Technology Co., Ltd. (Nanjing, China). Coumarin 6 (C6, ≥99% purity) and TPGS were purchased from Shanghai Aladdin Biochemical Technology Co., Ltd. (Shanghai, China). 1,4-dioxane, NAC, N, N’-carbonyldiimidazole (CDI), 1-hydroxybenzotriazole (HOBt), 1-ethyl-(3-dimethylaminopropyl) carbodiimide hydrochloride (EDC·HCl), and polyethylene glycol (PEG 20000) were all purchased from Shanghai Macklin Biochemical Co., Ltd. (Shanghai, China). The Caco-2 cell line and HT29-MTX-E12 cell line were purchased from Shanghai Fuheng Biotechnology Co., Ltd. (Shanghai, China). The MLg cell line was purchased from BeNa Culture Collection (BNCC, Xinyang, China).

### 2.2. Animals

Male Sprague–Dawley (SD) rats weighing 220 ± 20 g were sourced from Hangzhou Ziyuan Laboratory Animal Technology Co., Ltd., Hangzhou, China (Animal Production License: SCXK (Zhejiang) 2024-0004) and maintained at the Animal Experiment Center of Wannan Medical University. Prior to testing, all animals underwent a 7-day acclimatization period with unrestricted access to feed and water. The housing facility was kept at a constant temperature of 25 ± 5 °C and a relative humidity of 45 ± 5%.

### 2.3. Synthesis of TPGS-NAC Carrier Material

TPGS derivatives were synthesized via a three-step route [36]. In the first step, 614 mg TPGS and 384 mg CDI were dissolved in 1,4-dioxane, and the mixture was stirred at 37 °C for 14 h. The crude mixture was transferred into a dialysis bag (MWCO 1000 Da) and dialyzed against deionized water at room temperature under stirring for 48 h. After freeze-drying, TPGS-CDI intermediate was harvested. In the second step, 260 mg TPGS and 62.5 mg EDC·HCl were separately dissolved in DMSO. The solution was stirred at 25 °C in a nitrogen atmosphere for 24 h, followed by dialysis and freeze-drying to yield TPGS-NH_2_. TPGS-NAC conjugates were synthesized using the HOBt/EDC·HCl catalytic system. Briefly, 320 mg TPGS-NH_2_ and 60 mg HOBt were dispersed in anhydrous ethanol and treated by sonication. Separately, 64 mg NAC and 50 mg EDC·HCl were dissolved in anhydrous ethanol, and this solution was slowly added to the TPGS-NH_2_ dispersion. The pH of the mixed solution was adjusted to 5.0, and the amidation reaction proceeded under light protection for 3 h. The reaction mixture was further dialyzed against 5 mmol/L HCl solution at 4 °C in the dark. The purified dispersion was freeze-dried to obtain TPGS-NAC.

### 2.4. Verification of the TPGS-NAC Carrier Material

#### 2.4.1. Fourier Transform Infrared Spectroscopy Analysis

Fourier-transform infrared spectroscopy (FTIR) was carried out using the KBr pellet method. Different samples, including TPGS, NAC, the physical mixture of TPGS/NAC, and TPGS-NAC, were taken and mixed thoroughly with dry KBr powder for pellet formation. The infrared spectra were collected using a Fourier-transform infrared spectrometer in the wavenumber range of 4000–400 cm^−1^ with a resolution of 4 cm^−1^. By comparing the appearance, disappearance and shift changes of the characteristic absorption peaks of each sample, the chemical bond interaction between TPGS and NAC was analyzed.

#### 2.4.2. X-Ray Diffraction

The freeze-dried powders of NAC, TPGS, and TPGS-NAC were weighed separately, and X-ray diffraction (X-RD) analysis was conducted on each of them. Among them, the scanning angle range 2θ was from 5° to 50°, and the scanning rate was 2° per minute.

#### 2.4.3. Nuclear Magnetic Resonance Hydrogen Spectrum

We separately weighed the freeze-dried powders of NAC, TPGS, TPGS-NH_2_, and TPGS-NAC. The samples were all dissolved in deuterated dimethyl sulfoxide (DMSO-d_6_), and the liquid nuclear magnetic hydrogen spectra were recorded using a nuclear magnetic resonance spectrometer [37].

### 2.5. Preparation of IPT@NAC-TFS Micelles

In this experiment, 30 mg of TPGS-NAC, 60 mg of Pluronic^®^ F108, 30 mg of Lipoid^®^ S-100 and 9 mg of IPT were precisely weighed and placed in a 100 mL round-bottom flask. All solid powders were fully dissolved in 80 mL of absolute ethanol. A uniform thin drug-carrier film was formed on the inner wall of the flask after vacuum rotary evaporation at 40 °C to completely remove the organic solvent. Afterward, 10 mL of ultrapure water together with three 1 cm diameter glass beads was supplemented into the flask. The residual film was sufficiently hydrated through 15 min of rotary evaporation at a rotating speed of 150 rpm. After complete dissolution, the mixture was treated with ultrasonic dispersion for 10 min to obtain a uniform solution. A 0.22 μm PTFE syringe filter was used to filter the final dispersion, which yielded homogeneous IPT@NAC-TFS micelles. For control preparation, blank IPT@TFS micelles were fabricated using the identical protocol, where TPGS-NAC was substituted with equivalent TPGS while all other experimental conditions were kept consistent. Both micelle samples were preserved at 4 °C under dark conditions for subsequent tests.

### 2.6. Characterization of IPT@NAC-TFS Micelles

The optical Tyndall phenomenon of freshly prepared IPT@NAC-TFS micelles was visually observed. A Litesizer™ 500 particle analyzer (Anton Paar, Graz, Austria) was employed to determine the particle size distribution and zeta potential of the micellar samples. For transmission electron microscopy (TEM) observation, the micelle solution was diluted to a suitable concentration. A drop of diluted sample was loaded onto a copper grid and naturally dried at room temperature. The sample grid was negatively stained with 2% (*w*/*v*) phosphotungstic acid solution for 30 s before microscopic imaging. The microscopic morphology of micelles was finally captured using a Tecnai G2 Spirit transmission electron microscope (FEI Company, Hillsboro, OR, USA).

### 2.7. Determination of Critical Micelle Concentration for IPT@NAC-TFS Micelles

The pyrene fluorescence probe method was adopted to measure the CMC of the hybrid micelles. Briefly, an appropriate volume of 7.5 × 10^−5^ mol/L pyrene-acetone solution was added to brown volumetric flasks. Pure nitrogen was pumped into the flasks to completely volatilize acetone solvent, followed by the addition of blank micelle aqueous solutions. After constant volume adjustment, all samples were subjected to 40 kHz ultrasonication for 30 min in dark environments and incubated overnight to achieve full embedding of pyrene molecules into micelle structures, with the final pyrene concentration maintained at 7.5 × 10^−7^ mol/L. Fluorescence scanning was performed via an F-4600 fluorescence spectrophotometer (Hitachi, Tokyo, Japan) at a fixed excitation wavelength of 334 nm. The fluorescence emission values at 373 nm and 384 nm were recorded with a slit width of 2.5 nm. The CMC value of the micelles was calculated based on the changing trend of the fluorescence intensity ratio of the two characteristic peaks.

### 2.8. Establishment of In Vitro Analytical Method for IPT

Quantitative detection of IPT was performed using an Agilent 1290 ultra-high-performance liquid chromatography (UPLC) system (Agilent Technologies, Santa Clara, CA, USA), and the detection wavelength was set at 300 nm. Chromatographic separation was achieved on an InfinityLab Poroshell 120 EC-C18 column (100 mm × 2.1 mm, 2.7 μm, Agilent Technologies, USA). The column oven temperature was stabilized at 30 °C, and the sample injection volume was fixed at 10 μL. Methanol and ultrapure water were adopted as mobile phases A and B, respectively, with a constant flow rate of 0.3 mL/min. The gradient elution program was set as follows: 0–2 min, 55% phase A; 2–4 min, linear elevation of phase A proportion from 55% to 80%; 4–7 min, sustained at 80% phase A.

To construct the standard calibration curve, pure IPT reference substance was precisely weighed and fully dissolved in methanol via ultrasonic treatment. After constant volume configuration, the primary standard stock solution was obtained. A set of working standard solutions with serially diluted concentrations were prepared and measured under the aforementioned UPLC conditions. The standard curve was fitted by linear regression with IPT concentration as the horizontal coordinate and corresponding peak area as the vertical coordinate. For micelle sample pretreatment, 100 μL of micelle dispersion was mixed with 9-fold volume of methanol. The mixture was ultrasonicated for 10 min to achieve complete demulsification, followed by filtration through a 0.22 μm microporous membrane. The resulting filtrate was collected as the test solution for subsequent UPLC quantitative analysis.

### 2.9. Determination of Encapsulation Efficiency and Drug Loading Capacity

Ultrafiltration centrifugation was utilized to measure the encapsulation efficiency (EE) and drug loading (DL) of the fabricated micelles. In specific operations, 0.5 mL of freshly prepared micelle dispersion was added to ultrafiltration centrifugal tubes with a molecular weight cutoff of 1000 Da, followed by centrifugation at 13,000 rpm for 15 min. The filtrate containing unencapsulated free IPT was harvested and subjected to UPLC detection to quantify the mass of free drug (W_F_). Meanwhile, an equal volume of micelle sample was diluted 10 times with methanol and treated with 10 min ultrasonication to thoroughly break the micelles’ structure. After filtering through a 0.22 μm microporous membrane, the total amount of IPT (W_T_) in the whole delivery system was determined via UPLC analysis. Finally, the EE and DL of micelles were calculated separately based on the established Equations (1) and (2), respectively.

(1) $$\mathrm{EE}\%=\frac{W_{T}-W_{F}}{W_{A}}\times 100\%\;$$

(2) $$\mathrm{DL}\%=\frac{W_{T}-W_{F}}{W_{M}}\times 100\%\;$$

In the equations, W_T_ denotes the mass of drug released following demulsification treatment, W_F_ represents the mass of free drug, W_A_ indicates the initial total drug dosage, and W_M_ signifies the total mass of carrier material and drug within the micellar system.

### 2.10. In Vitro Release Experiment

The in vitro release profile of IPT from different formulations was assessed using a conventional dialysis technique. To establish sustainable sink conditions for hydrophobic IPT release, pH 6.8 PBS buffer supplemented with 1% Tween 80 was adopted as the release medium. In this procedure, 1 mL of IPT suspension, IPT@TFS micelles and IPT@NAC-TFS micelle samples (all standardized to an identical IPT concentration of 400 μg/mL) were individually loaded into dialysis bags with a molecular weight cutoff ranging from 8000 to 14,000 Da. After tight sealing, the dialysis bags were completely submerged in the release medium and incubated in a constant-temperature shaking incubator at 37 °C with a shaking rate of 100 rpm. At scheduled time intervals of 0.5, 1, 2, 4, 6, 8, 12 and 24 h, 1 mL of release medium was collected for subsequent quantitative analysis. The IPT concentration in withdrawn samples was determined by UPLC following the chromatographic parameters illustrated in Section 2.8. Equation (3) was used to calculate the cumulative drug release rate, and the release percentage versus time curve was finally plotted:

(3) $$\mathrm{Cumulative}\;\mathrm{release}\%=\frac{M_{R}}{M_{L}}\times 100\%\;$$

M_R_ and M_L_ represent the cumulative drug release and the total drug loading.

### 2.11. Mucus and Intestinal Epithelial Penetration of IPT@NAC-TFS Micelles

#### 2.11.1. Cellular Uptake

Exponentially proliferating Caco-2 and HT29-MTX-E12 cells were co-seeded in confocal culture dishes at a 7:3 ratio and incubated overnight for adequate cell adhesion, following an experimental scheme reported previously [38]. Coumarin 6 (C6) was used as a fluorescent label to synthesize two C6-loaded micellar formulations: C6@TFS and C6@NAC-TFS micelles. We treated intact cell monolayers with serum-free DMEM supplemented with free C6, C6@TFS micelles or C6@NAC-TFS micelles over a 2 h incubation period. The overall C6 content was normalized to 0.5 μg/mL across all groups to exclude the influence of inconsistent C6 dosage on cellular internalization behavior. Cells were rinsed three times with PBS post-incubation, prior to fixation. Samples were fixed in 4% paraformaldehyde solution for 15 min, and nuclear compartments were stained with 10 μg/mL Hoechst 33342 for 10 min to observe nuclear profiles. Fluorescent confocal images were acquired utilizing a TCS SP8 laser scanning confocal microscope (Leica Microsystems, Wetzlar, Germany) fitted with a 63× oil immersion objective (Leica, Wetzlar, Germany). Nucleus blue fluorescence was detected at λ_ex_ = 405 nm/λ_em_ = 465 nm, and green C6 fluorescence was recorded at λ_ex_ = 488 nm/λ_em_ = 568 nm. Merged images were analyzed to evaluate intracellular distribution and transmucosal uptake of micellar carriers.

#### 2.11.2. Investigation of Cellular Uptake Mechanisms

Caco-2 and HT29-MTX-E12 cells at logarithmic growth phase were seeded in 6-well plates at a 7:3 cell ratio and cultured for 24 h. Cells were pretreated for 1 h with different endocytosis inhibitors [39]: 20 μM indomethacin, 5 μM nystatin, 20 μM β-carotene, 1 mM protamine sulfate, 30 μM chlorpromazine and 1 mM sodium azide. The control group was treated with blank medium. After pretreatment, cells were incubated with C6@TFS or C6@NAC-TFS micelles in incomplete DMEM for 2 h. Subsequently, cells were trypsinized, washed three times with pre-cooled PBS, resuspended in 0.5 mL PBS, kept on ice in the dark, and analyzed using the FACSVerse flow cytometer (BD Biosciences, San Jose, CA, USA).

#### 2.11.3. Bidirectional Transport Across Cell Monolayers

Caco-2 mixed with HT29-MTX-E12 cells (7:3 proportion) in the exponential growth phase were plated onto 12-well Transwell polycarbonate inserts (3.0 μm pore size, 1.13 cm^2^ effective growth area; NEST Biotechnology Co., Ltd., Wuxi, China). Cells were cultured continuously for 21 days to build integrated epithelial barriers. We monitored monolayer barrier integrity via TEER measurement using an EVOM2 epithelial volt–ohm meter (World Precision Instruments, Sarasota, FL, USA). Only inserts with TEER readings exceeding 400 Ω·cm^2^ were retained for subsequent transmembrane permeation experiments. Prior to permeation testing, old culture medium was aspirated, and cell monolayers were rinsed three times with HBSS, followed by a 30 min equilibration at 37 °C. For the apical-to-basolateral (AP→BL) transport direction, 500 μL HBSS containing free IPT, IPT@TFS micelles or IPT@NAC-TFS micelles was added to the apical chamber, while 1.5 mL blank HBSS filled the basolateral compartment. For basolateral-to-apical (BL→AP) permeation, 1.5 mL drug-spiked HBSS was placed in the basolateral chamber, with 500 μL blank HBSS loaded in the apical side. The Transwell plates were incubated at a constant temperature of 37 °C. After 4 h of incubation, 500 μL samples were collected from both chambers individually. Collected specimens were centrifuged at 12,000 rpm for 10 min, and UPLC was used to measure IPT concentrations in the resulting supernatants. Calculation of the apparent permeability coefficient (P_app_) was performed based on Equation (4):

(4) $$P_{\mathrm{app}}=\frac{V}{S\times C}\times \frac{\mathrm{dC}}{\mathrm{dt}}$$

where dc/dt denotes the rate of change of drug concentration per unit time; S represents the membrane surface area (1.13 cm^2^); C denotes the initial drug concentration; and V denotes the volume of the receiving solution.

### 2.12. IPT@NAC-TFS Micelles for Attenuating Oxidative Stress Injury in MLg Mouse Fibroblasts

#### 2.12.1. In Vitro Cell Viability Assay After Drug Treatment

We harvested MLg fibroblasts in the exponential growth phase and prepared single-cell suspensions via digestion using 0.25% trypsin–EDTA solution. The cell suspension was diluted with complete DMEM medium and evenly distributed into 96-well plates at a volume of 100 μL per well. These culture plates were placed in a 37 °C incubator supplemented with 5% CO_2_ and cultured for 24 h until cell coverage reached roughly 90%. Cells were then exposed to gradient concentrations of IPT@TFS micelles and IPT@NAC-TFS micelles over a 24 h treatment period. Cells maintained in drug-free incomplete culture medium were designated as the blank control group. Upon completion of drug incubation, 10 μL of CCK-8 working solution was supplemented into every well. After a 2 h shading incubation at 37 °C, we recorded the OD value at the 450 nm wavelength using a microplate spectrophotometer, which was further used to compute the cell viability percentage for all test groups.

Using an identical procedure, the half-maximal inhibitory concentration (IC_50_) of bleomycin (BLM) toward MLg cells after 48 h incubation was determined to confirm the concentration for cell injury model construction. To explore the protective effects of free IPT and micellar formulations against BLM-induced damage, MLg cells were seeded into 96-well plates and divided into six groups: control group, BLM model group (10 μM), positive control group (1000 μM NAC) [40], free IPT group, IPT@TFS micelles group, and IPT@NAC-TFS micelles group (7 μM IPT). Except for those in the control group, all cells were exposed to BLM for 24 h to establish the oxidative damage model. After model establishment, the medium was replaced. The model group received continuous BLM stimulation for another 24 h. For treatment groups, medium containing BLM together with corresponding test formulations was added, and incubation continued for 24 h. Cell viability was finally evaluated via the CCK-8 assay under consistent culture and detection conditions.

#### 2.12.2. Intracellular ROS Measurement

MLg cells were seeded into 6-well plates and cultured for 24 h until cell confluence reached about 90%. Cell injury modeling and drug administration were performed following the same grouping protocol described above. After treatment, cells were rinsed with PBS, trypsinized and harvested. Residual trypsin was removed by resuspension and washing with pre-cooled PBS. Cells were incubated with 10 nmol/mL DCFH-DA solution for 30 min. After PBS washing, intracellular ROS levels were quantified by flow cytometry [41].

#### 2.12.3. Determination of Oxidative Stress Levels in MLg Cells

MLg cells were seeded into six-well culture plates and cultured for 24 h to allow cell attachment. The cell injury model establishment and subsequent drug intervention were performed following the experimental procedures described above. Once the drug treatment process was completed, cell monolayers were rinsed using PBS buffer, harvested via gentle scraping, and pelleted through centrifugation. We resuspended the collected cell sediments in pre-cooled PBS solution and broke cells by sonication within an ice bath to prepare total cell homogenate samples. After calibrating the total protein concentration of every homogenate sample, we tested SOD activity and MDA levels strictly in line with the operating manuals supplied by matched commercial detection kits [42].

### 2.13. Pharmacokinetic Study

Fifteen male Sprague–Dawley (SD) rats weighing 220 ± 20 g were split into three groups randomly (*n* = 5). Animals were deprived of food for 12 h overnight before drug delivery, with free access to drinking water throughout the fasting period. Three test preparations, including free IPT suspended in 0.5% CMC-Na solution, IPT@TFS micelles and IPT@NAC-TFS micelles, were given by intragastric administration at an equivalent IPT dose of 100 mg/kg. We collected roughly 200 μL venous blood from the retro-orbital plexus at preset time points after gavage: 5 min, 15 min, 30 min, 60 min, 90 min, 2 h, 3 h, 4 h, 6 h, 8 h, 12 h and 24 h. Blood samples were immediately transferred into heparin-treated tubes and centrifuged at 3500 rpm for 10 min to separate plasma components. During plasma sample pretreatment, 100 μL plasma was mixed sequentially with 20 μL osthole internal standard solution (40 μg/mL), 50 μL 0.2% Triton X-100 and 830 μL methanol. After thorough vortex mixing and centrifugation, the supernatant was collected and fully dried under vacuum. The dry residue was re-dissolved with a small amount of methanol for subsequent UPLC measurement [43,44]. Major pharmacokinetic parameters were calculated using DAS 2.0 software. Quantitative detection of plasma imperatorin was performed on an Agilent 1290 UPLC system fitted with a UV detector set at 300 nm, with an injection volume of 10 μL. Chromatographic separation was carried out on a Boltimate-C_18_ column (2.1 × 100 mm, 2.7 μm) at a constant flow rate of 0.3 mL·min^−1^ and a stable column temperature of 30 °C. Water (mobile phase A) and methanol (mobile phase B) constituted the mobile phase, with the following gradient elution schedule: 0–1.5 min, 55% A; 1.5–5.5 min, linear gradient to 70% A; 5.5–6.5 min, linear gradient to 80% A; 6.5–6.6 min, linear gradient to 88% A; and 6.6–10 min, maintained at 88% A. Calibration curves were plotted with IPT concentrations as the horizontal coordinate and the peak area ratio of IPT to osthole internal standard (IS) as the vertical coordinate.

### 2.14. Intestinal Distribution in Rats

Rats received oral administration of free C6 solution, C6@TFS micelles and C6@NAC-TFS micelles at an equivalent C6 dose of 10 mg/kg. Two hours following oral delivery, all animals were sacrificed, and intestinal tissues were immediately dissected out. The harvested intestinal segments were washed using pre-cooled physiological saline, subjected to frozen section treatment and sliced into 5 μm thick slices before being affixed onto glass slides. After 10 min of DAPI nuclear staining, these tissue slices were visualized and photographed with a laser scanning confocal microscope.

### 2.15. Statistical Analysis

All experimental assays were conducted in technical triplicates alongside three independent biological repeats. All measured data are presented as the mean ± standard deviation (SD). Statistical analyses were implemented with SPSS 17.0 software package. Intergroup comparisons were performed via Student’s *t*-test. Any observed differences with *p* values below 0.05 were regarded as statistically meaningful.

## 3. Results and Discussion

### 3.1. Characterization of the TPGS-NAC Carrier Material

**Figure 1:**
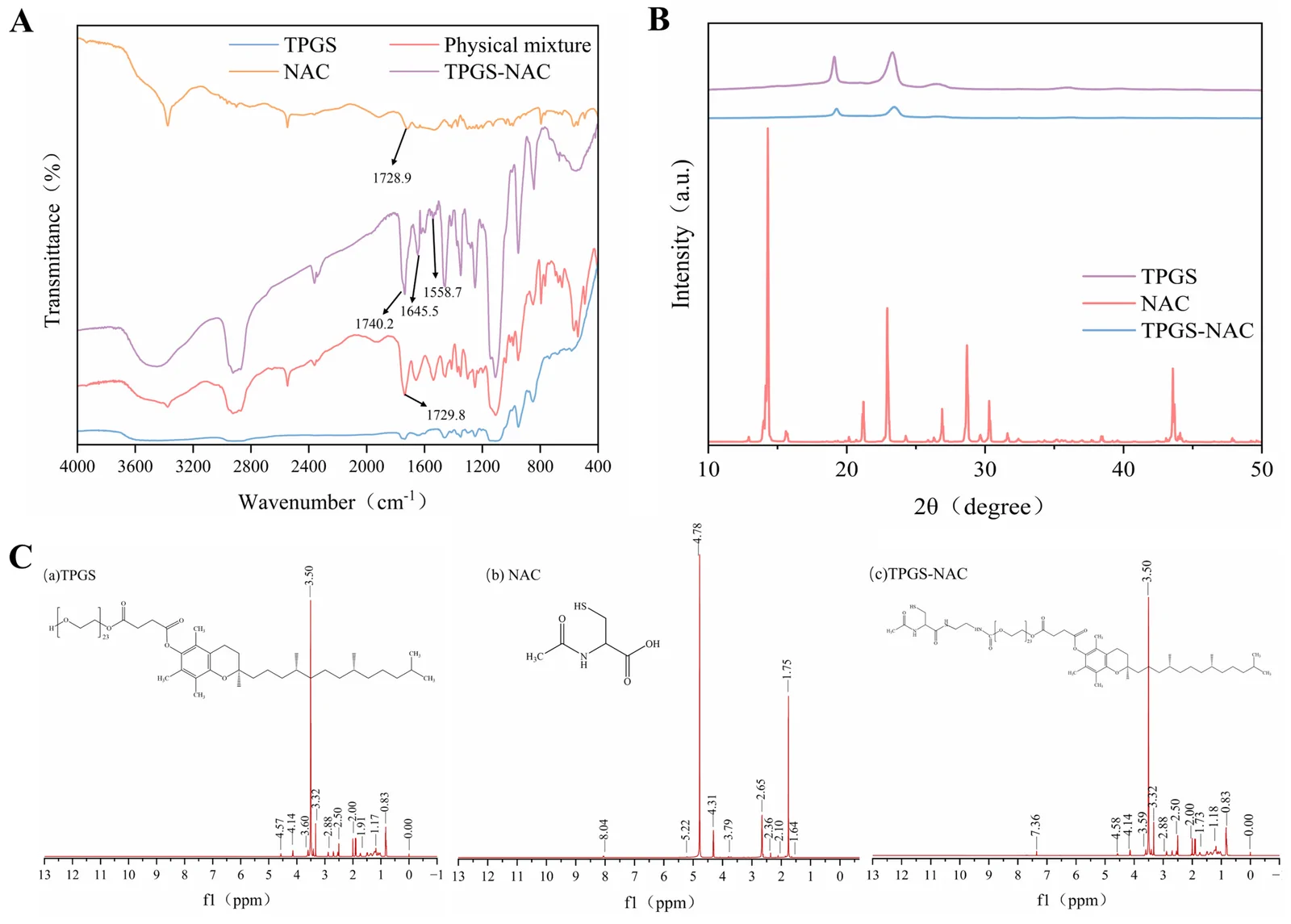
Synthesis and characterization of TPGS-NAC. FTIR spectra of TPGS, NAC, their physical mixture, and the TPGS-NAC (**A**). XRD patterns of TPGS, NAC, and the TPGS-NAC (**B**). ^1^H NMR spectrum of TPGS, NAC, TPGS-NAC (**C**).

Figure 1A presents the FTIR data of TPGS, NAC, their mechanical blend, and TPGS derivatized with NAC (TPGS-NAC). TPGS-NAC has a new specific peak at 1645.5 cm^−1^ in the TPGS-NAC profile that corresponds to the amide I band and is absent in the TPGS profile. At the same time, a signal at 1558.7 cm^−1^ is also an amide II group [45]. It should be noted that the characteristic carboxyl stretching peak of NAC at 1728.9 cm^−1^ is no longer present in the TPGS-NAC profile, and in its place, a strong ester carbonyl peak at 1740.2 cm^−1^ has appeared. On the other hand, the simple mechanical blending still had the original carboxyl band of NAC at 1729.8 cm^−1^. Therefore, there is a direct link between NAC and the TPGS structure.

The XRD patterns of unmodified TPGS, isolated NAC and the synthesized TPGS-NAC are shown in Figure 1B; that is, the scattering intensity as a function of 2θ is displayed. Free NAC has many sharp and strong diffraction peaks, so it is a highly ordered crystal. TPGS is an amphiphilic polymer and shows only a faint, broad diffraction peak; thus, it is in an amorphous state [46]. After chemical coupling, the obtained TPGS-NAC product had a noticeable decrease and broadening of the NAC crystal peaks, and some specific diffraction signals disappeared, but many of the characteristic TPGS diffraction signs were still present. Therefore, the above results show that covalent functionalization has successfully broken the ordered crystal lattice of NAC and made the prepared conjugate less crystalline.

According to the systematic analysis of the ^1^H-NMR hydrogen spectra, there is a noticeable methylene signal at 3.5 ppm corresponding to the PEG group of TPGS, and the alkyl chain of vitamin E is in the 0.8–1.5 ppm region. NAC has an acetyl group at 2.1 ppm, and methylene protons adjacent to the amino and carboxyl groups are in the 4.0–4.5 ppm range in the ^1^H-NMR spectrum. The broad amide proton signal in the 6.0–8.5 ppm region of the new TPGS-NAC sample shows that amide bonds have been formed, and at 4.1 ppm, there is a specific methylene peak corresponding to the ester bond [47]. The conservation of the structure of TPGS also indicates that NAC can form a covalent bond with the amino group of TPGS (Figure 1C). Therefore, the preparation of TPGS-NAC has been confirmed by various means such as ^1^H-NMR, X-RD and FTIR.

### 3.2. Preparation and Characterization of IPT@NAC-TFS Micelles

**Figure 2:**
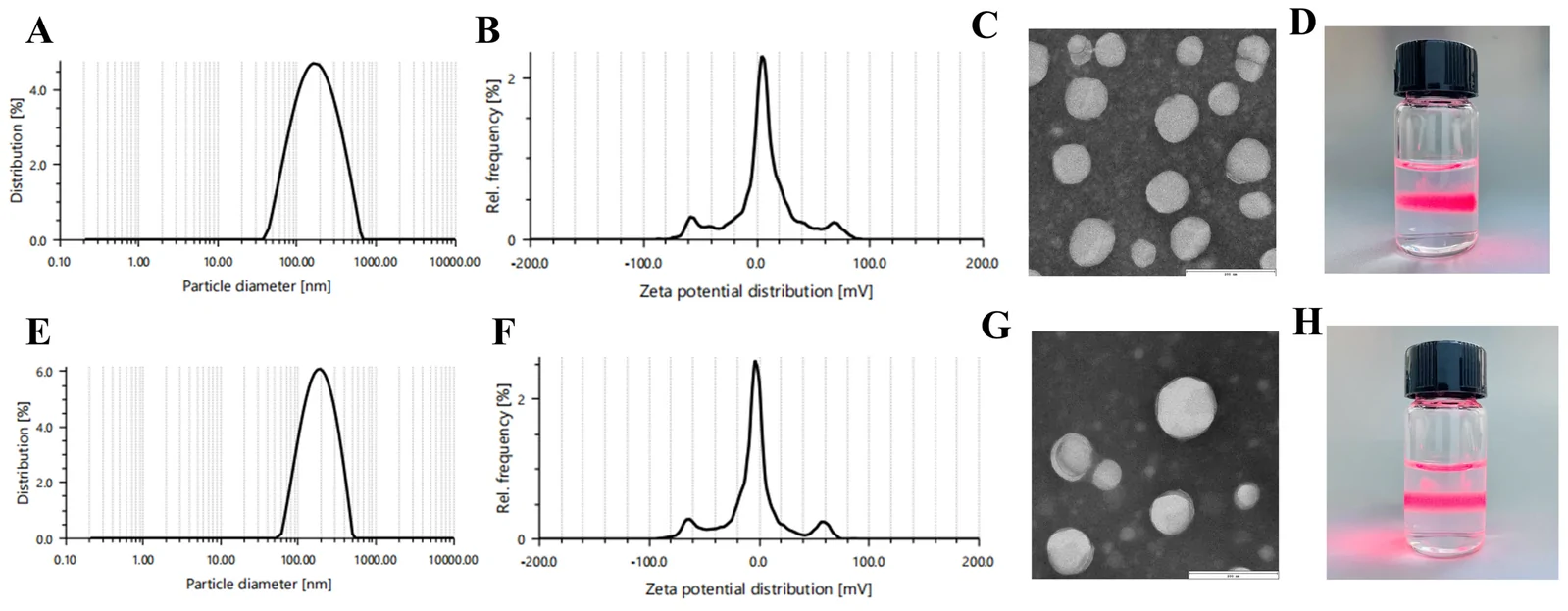
Preparation and characterization of micelles. Size distribution (**A**), zeta potential distribution (**B**), TEM image (**C**) and appearance (**D**) of IPT@TFS micelles. Size distribution (**E**), zeta potential distribution (**F**), TEM image (**G**) and appearance (**H**) of IPT@NAC-TFS micelles. (mean ± SD, *n* = 3).

The size, polydispersity index (PDI) and zeta potential of the micelle were analyzed to confirm that it had formed properly and how well it disperses. The mean hydrodynamic radius of the unmodified IPT@TFS micelles was 102.72 ± 2.08 nm, the PDI was 0.234 ± 0.015, and the almost flat surface charge was 1.40 ± 0.10 mV (Figure 2A,B). NAC grafting increased the particle size to 117.63 ± 3.65 nm and reduced the PDI to 0.225 ± 0.008; however, the zeta potential was about −2.60 ± 0.30 mV (Figure 2E,F) and was still close to electric neutrality. Therefore, it was concluded that both types of micelle systems exhibit good monodispersity. A small increase of 15 nm in the size of the particles and a slight reversal of the surface charge were also observed after the addition of NAC groups. The change in surface electrostatic characteristics is due to the addition of many free carboxyl groups from NAC modification on the TPGS backbone. Ionizable carboxyl groups are in the form of ions in water-based solutions, so they add a weak negative surface charge to the modified micelles and change the small positive charge of unmodified IPT@TFS micelles. In addition, both populations of micelles had almost no surface charge and, thus, could improve the permeability of the intestinal wall and avoid non-specific binding in vivo during transport; therefore, there was less inflammation of the intestinal mucosa.

To observe the fine structure of many different micelles, transmission electron microscopy was employed. As shown in Figure 2C,G, both IPT@TFS micelles and IPT@NAC-TFS micelles had a uniform, well-ordered spherical shape and did not show any obvious aggregation. Note that the size of the particles in TEM was slightly smaller than that of hydrated particles in a water-based solution. The main cause of the change is the drying and shrinkage of the hydrated outer shell of the micelles during the drying of the sample. Based on visual observation of the dispersions of the micelles, both are clear and show a distinct Tyndall effect in the presence of red laser light (Figure 2D,H); therefore, it can be determined that a stable colloidal micelle system has been formed.

### 3.3. Determination of Critical Micelle Concentration

**Figure 3:**
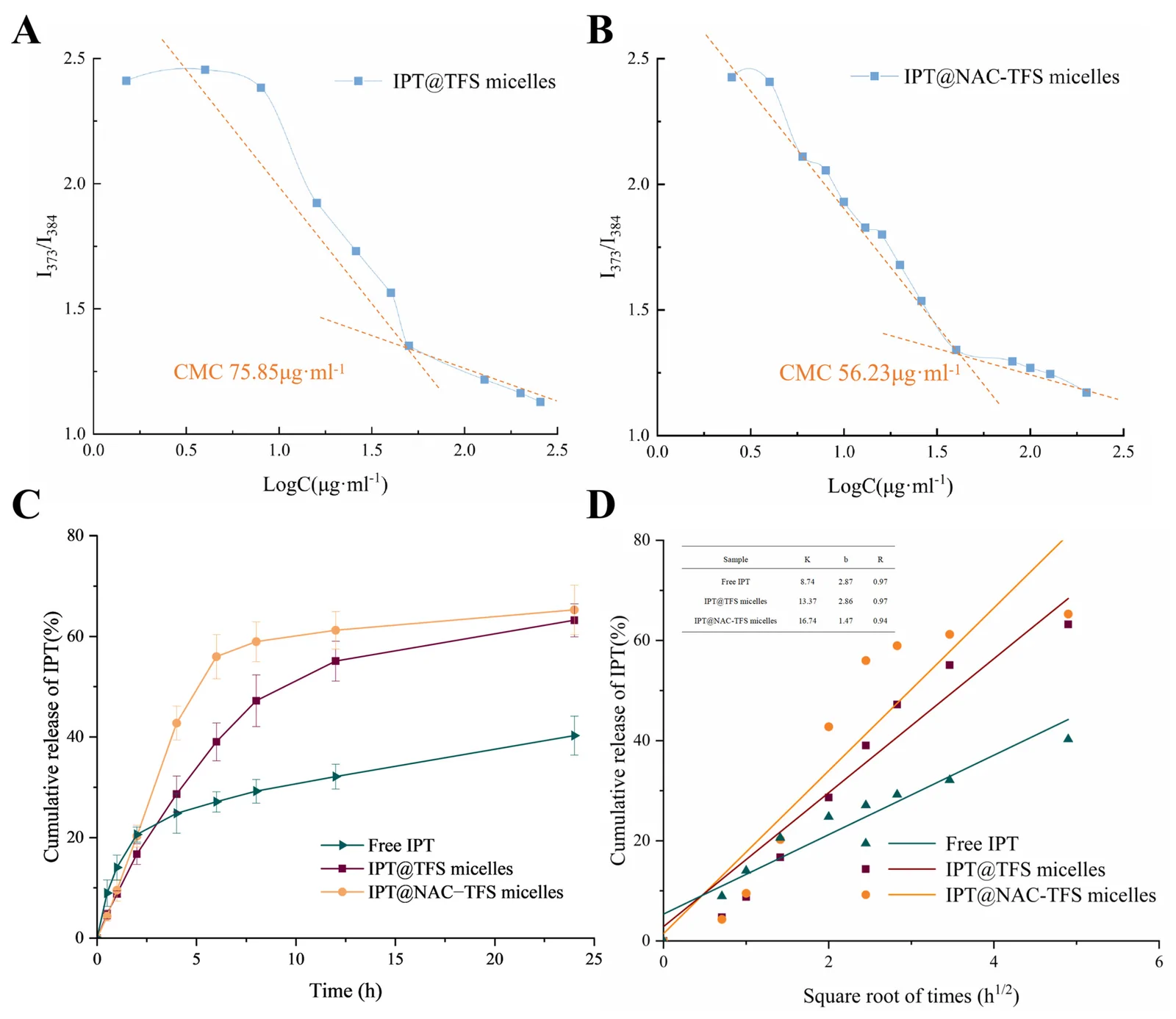
The characteristics of the micelles and drug release kinetics of IPT. CMC of IPT@TFS micelles (**A**), CMC of IPT@NAC-TFS micelles (**B**). The in vitro release of IPT (**C**). Higuchi model fitting for IPT release (**D**). (mean ± SD, *n* = 3).

The CMC of the mixed micelles was evaluated using the pyrene fluorescence probe method. In the graph of analysis, the logarithmic concentration of the micelles is shown on the horizontal axis, and the fluorescence intensity ratio of pyrene at 373 nm and 384 nm is plotted on the vertical axis. Based on the fitting results, it was determined that the CMC values of the IPT@TFS micelles and IPT@NAC-TFS micelles were 75.85 μg/mL and 56.26 μg/mL, respectively (Figure 3A,B). A lower CMC indicates stronger intermolecular hydrophobic interactions, which enables the micelles to maintain their structural integrity under gastrointestinal dilution conditions and reduces the likelihood of dissociation. The CMC of the NAC-coated micelles was lower than that of the unmodified IPT@TFS micelles; thus, it could be determined that NAC coupling increased the density of copolymer segments in the micellar core. The first reason for this is that the hydrophilic–hydrophobic balance of the surface has changed after modification. Much digestive fluid will be present in the gastrointestinal tract and, thus, dilute the micelles. IPT@NAC-TFS micelles have a relatively small CMC value, good dilution resistance and thermodynamic stability; thus, they can remain in the mixed micelles for a long time in the intestine and avoid the early release of the encapsulated IPT drug.

### 3.4. Encapsulation Efficiency and Drug Loading Content

The linear range of the UPLC quantification method for IPT is 0.05–200.00 µg/mL. The model for the reference calibration plot is y = 59.32x − 6.0112, and the corresponding R = 0.9997. The drug loading content and encapsulation efficiency of the IPT@TFS micelles were 6.72 ± 0.28% and 99.52 ± 0.11%, respectively. For the IPT@NAC-TFS micelles, the corresponding values were 6.82 ± 0.16% and 99.67 ± 0.04%. No obvious differences in loading performance were observed between the two formulations, which reveals that NAC modification hardly interfered with IPT encapsulation. Micelles also have a good encapsulation efficiency and a stable drug loading ratio; thus, they will continue to be employed in the next phase of the research to improve oral bioavailability by crossing the intestinal epithelial layer.

### 3.5. In Vitro Release

Figure 3C displays the in vitro dissolution curve of the two micelle systems. Both mixed micelle formulations exhibited low drug release of approximately 20% within the initial two hours. The majority of the drug remained encapsulated within micelles, which protects the loaded drug and favors subsequent intestinal absorption. From a physiological perspective, oral formulations have a limited effective absorption window in the small intestine. Drugs are generally absorbed within several hours after oral administration, while unabsorbed fractions are transported to the large intestine and eventually excreted. After 6 h of incubation, the drug content in both groups of micelles increased, and the drug release rate also accelerated. The micelles exhibited sustained drug release in the release medium. The sustained release profile of the micelles within this physiological time window facilitates drug dissolution and further absorption into the bloodstream. In 24 h, the total release fractions of the IPT@TFS micelles and IPT@NAC-TFS micelles were 60.1 ± 0.4% and 61.4 ± 2.8%, respectively, and both of these values were significantly higher than the 37.2 ± 1.3% released by the free IPT solution. Therefore, it was found that micellar vehicles can extend the life of the drug and significantly improve the in vitro release efficiency of poorly soluble IPT.

To study the distribution of the release data more thoroughly, the Higuchi kinetic model was used to fit all of them (Figure 3D). The release curve of micelles’ IPT followed the Higuchi equation well; thus, it was concluded that drug diffusion was the main cause of release. The fit curve of the IPT@NAC-TFS micelles is much steeper than that of the others; therefore, it has a relatively high speed of internal diffusion for IPT. Both kinds of micelles have the same total amount of material release, but they are different in terms of their internal microenvironment; therefore, the diffusion coefficient of NAC-modified micelles is relatively large. To alter the degree of both the hydrophilicity and hydrophobicity of the conjugate NAC, polar carboxylic groups can be attached; thus, it is hoped that the rate of egress for lipophilic IPT molecules will be increased. The functionalization of NAC can increase the speed of drug release from the inside and maintain the extended release effect of micelles; therefore, fine and flexible control over drug release can be realized.

### 3.6. Cellular Uptake

**Figure 4:**
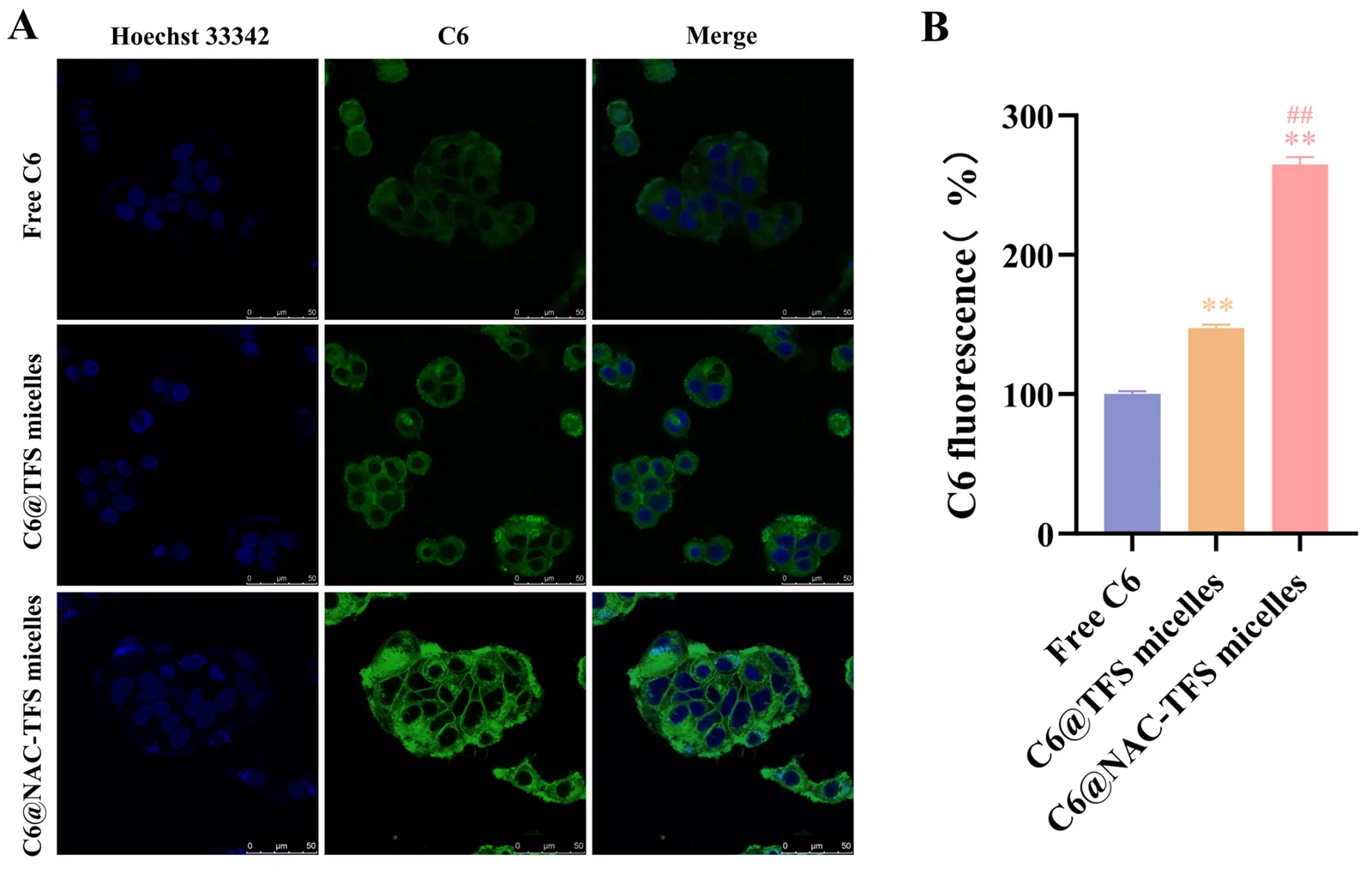
Study of the uptake of Caco-2/HT29-MTX-E12 cells. Uptake CLSM images of Caco-2/HT29-MTX-E12 co-cultured cells incubated with free C6, C6@TFS micelles and C6@NAC-TFS micelles (**A**). Quantitative fluorescence analysis of C6 uptake by free C6, C6@TFS micelles and C6@NAC-TFS micelles (**B**). ** *p* < 0.01 vs. free IPT. ^##^ *p* < 0.01 vs. IPT@TFS micelles. (mean ± SD, *n* = 3).

Caco-2 cells and HT29-MTX-E12 cells separately have the biological characteristics of intestinal enterocytes and goblet cells, respectively. A dual-cell co-culture system was employed to secrete mucus and mimic the microenvironment of the small intestinal epithelium. Therefore, this kind of structure has been applied many times to study the absorption characteristics of oral mixed micelles in a test tube. As shown in Figure 4A, the fluorescent marker C6 was green in the cytoplasm, and the cell nuclei were blue after Hoechst 33342 staining. According to the statistics of the fluorescence assessment, it was found that the cells contained a relatively large number of C6@TFS micelles and C6@NAC-TFS micelles compared to free C6 molecules (Figure 4B). Hydrophobic small-molecule entities, such as free C6, can enter cells by passive diffusion, but they are easily trapped and blocked by the dense meshwork of mucus and cannot be taken up by cells. Mixed micelles can also be taken up by epithelial cells through energy-consuming and invagination-based endocytosis to increase the amount of drug in the cell. C6@NAC-TFS micelles were taken up by the cells more than C6@TFS micelles. The main reason for this increase is the intrinsic mucolytic effect of the linked NAC. NAC is a good mucolytic agent that can also cut the disulfide bonds in mucin. NAC is on the surface of the mixed micelles; thus, it can locally change the structure of mucus and increase the mobility of mixed micelles in the mucus layer. NAC-modified mixed micelles are small in size and have a small, negative zeta potential; therefore, they are less repelled by negatively charged mucins and can enter the mucus. Due to the reasons mentioned above, more mixed micelles can cross the mucus membrane and reach the surface of adjacent epithelial cells to increase the efficiency of cellular uptake.

### 3.7. Investigation of Cellular Uptake Mechanisms

**Figure 5:**
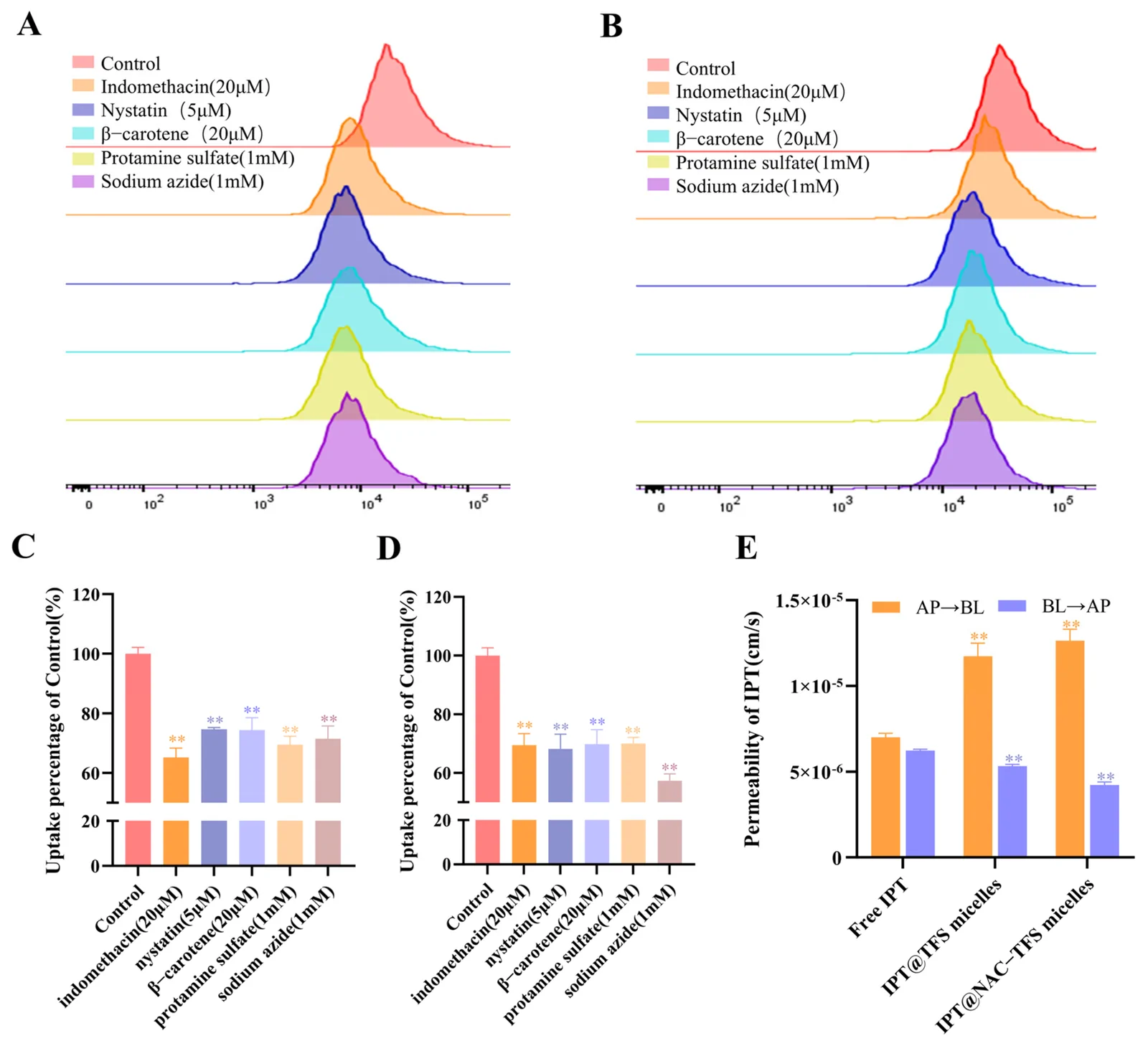
Research on the mechanism of Caco-2/HT29-MTX-E12 cell uptake and transport. FACS analysis of the effect of uptake inhibitors on C6@TFS micelles (**A**) and C6@NAC-TFS micelles (**B**). The effects of uptake inhibitors on the relative uptake rate of C6@TFS micelles (**C**) and C6@NAC-TFS micelles (**D**). AP→BL and BL→AP permeability of free IPT, IPT@TFS micelles and IPT@NAC-TFS micelles in transport study (**E**). ** *p* < 0.01 vs. free IPT. (mean ± SD, *n* = 3).

To determine how the micelles enter the Caco-2/HT29-MTX-E12 dual-layer culture, many inhibitors of endocytosis and transport were added. The graphs are the normalized uptake amounts after 60 min of administration of the above uptake inhibitors and then after 120 min of incubation with C6@TFS micelles (Figure 5A,C) or C6@NAC-TFS micelles (Figure 5B,D). Indomethacin is a drug that interferes with caveolae-based endocytosis [48]; nystatin affects lipid raft-dependent internalization [49]; β-carotene is a regulator of tight junctions [50]; protamine sulfate blocks adsorptive-mediated uptake [51]; and sodium azide inhibits cell metabolic energy and active transport [52]. Each inhibitor markedly decreased the cellular uptake of the mixed micelles, which indicates that multiple pathways are involved in cellular internalization. For both mixed micelle formulations, sodium azide, nystatin and indomethacin produced obvious inhibitory effects on cell uptake. These results suggest that the cellular internalization of mixed micelles is an energy-dependent process, predominantly mediated via caveolae and lipid raft-dependent endocytosis. NAC modification did not cause any change in the main endocytic pathway. Accordingly, the enhanced cellular uptake for NAC-TFS mixed micelles is attributed to improved mucus penetration capacity rather than alterations in cellular internalization mechanisms.

### 3.8. Transepithelial Transport

P_app_ is a general indicator of how much a substance can cross the intestinal lining through its cells, and it is used to assess this. According to the conventional classification norm for permeability, if the P_app_ rate is larger than 1 × 10^−6^ cm/s, it is classified as a high-permeability molecule; otherwise, if it is smaller than 1 × 10^−7^ cm/s, it is considered a low-permeability compound. The transepithelial transport profiles of the free IPT and micelle-based systems were studied by means of a model of a mucus-producing Caco-2/HT29-MTX-E12 dual-cell layer, and the corresponding permeability data are shown in Figure 5E. The P_app_ value of the free IPT in the apical to basolateral (AP→BL) direction was more than 1 × 10^−6^ cm/s, and, thus, it was determined that the permeability of the IPT molecules was high (Table 1) [53].

IPT@TFS micelles and IPT@NAC-TFS micelles increased the AP→BL Papp coefficient and reduced the BL→AP Papp compared to free IPT. Thus, it was determined that they could inhibit drug efflux. All of the above changes were statistically significant at the 0.01 level (*p* < 0.01), and, therefore, it can be concluded that the engineered mixed micelles have enhanced the transepithelial transport efficiency of IPT. The permeability of AP→BL for the IPT@NAC-TFS micelles group was relatively high, and the efflux potential of BL→AP was also low compared with that of the unmodified IPT@TFS micelles (*p* < 0.01). From the above, it can be concluded that grafting onto the NAC surface increases the total permeability of mixed micelles in the mucin-rich gut environment.

The efflux ratio is a relative quantity; thus, it can be employed to assess the type of transport across cells. According to the above reference values, a ratio of more than 2 or less than 0.5 is considered to reflect active transport-driven transepithelial transport; otherwise, it is thought that passive diffusion is the main mode of transport [54,55]. Among them, the free IPT had an efflux ratio of 0.5–2 and was therefore regarded as passively transported in the gut. At the same time, both kinds of micelles had an efflux ratio of less than 0.5 and, thus, were mainly taken up by enterocytes through active endocytosis, not passive transmembrane diffusion.

TPGS can be added to the two micelles above to provide good P-gp inhibition. After entering the cell, the released TPGS can inhibit the activity of the apical P-gp protein to prevent the efflux of intracellular IPT and, thus, reduce BL→AP drug permeability and increase net absorptive delivery. IPT in the micellar droplets is still in the free small-molecule form after transmembrane transport. The internalization of whole micelles via endocytosis further reduces the direct exposure of IPT to efflux transporters, generating a synergistic effect. NAC has the attribute of mucolytic activity, and, therefore, IPT@NAC-TFS micelles penetrate mucus more efficiently and arrive at epithelial surfaces in greater quantities. Therefore, based on the above reasons, it is expected that TPGS can inhibit P-gp and enhance the transepithelial permeability of NAC-modified micelles compared with non-modified micelles.

### 3.9. Oxidative Stress Experiment

#### 3.9.1. Cytotoxicity Evaluation

**Figure 6:**
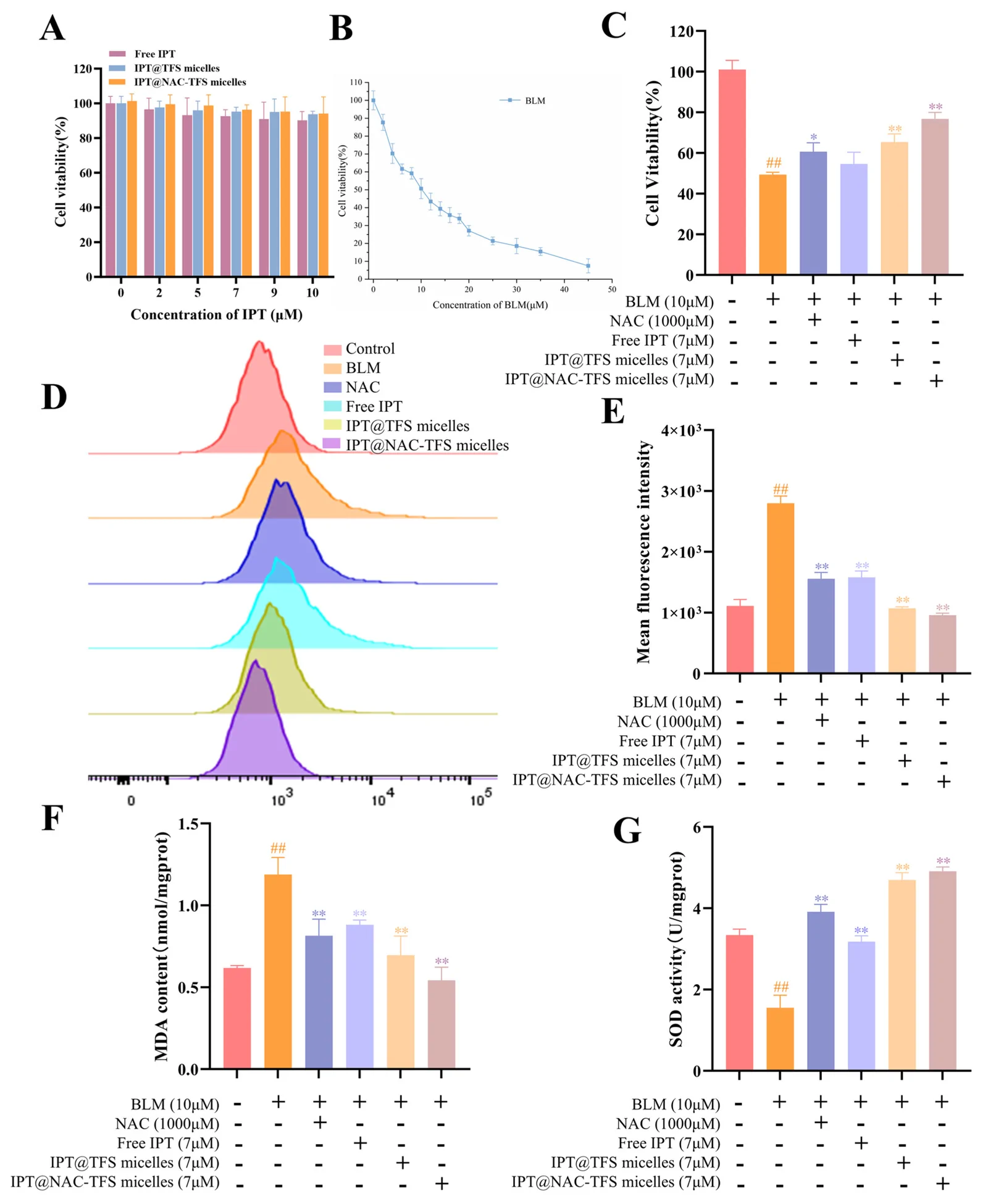
Oxidative stress experiments of MLg cells. The effects of different concentrations of IPT in free IPT, IPT@TFS micelles and IPT@NAC-TFS micelles on cell viability (**A**). The effects of different concentrations of BLM on cell viability (**B**). Effects of co-incubation with BLM on the viability of MLg cells in each treatment group. (**C**). FACS images of ROS in MLg cells (**D**). Quantitative analysis of fluorescence intensity in MLg cells (**E**). MDA content (**F**) and SOD activity (**G**) as indicators of oxidative stress. ^##^ *p* < 0.01 vs. control. * *p* < 0.05, ** *p* < 0.01 vs. BLM. (mean ± SD, *n* = 3).

Figure 6A shows the survival rate of MLg cells after 24 h of exposure to different concentrations of free IPT, IPT@TFS micelles and IPT@NAC-TFS micelles. The experimental data show that the cell viability in all treatment groups was more than 90% at every point in the full concentration test. Based on the above cell viability data, it can be concluded that the free IPT parent compound and both developed micellar systems are not toxic to the cells. Based on the current experimental conditions, none of the specimens have shown visible cytotoxicity in MLg cells; therefore, it is possible to conduct the next round of tests on antioxidant effects.

Bleomycin (BLM) is often used to form fibrotic damage frames in pulmonary cell cultures [56]. Figure 6B shows the viability of cells after 48 h of BLM treatment. A dose of 10 μM BLM was chosen to reduce the number of living cells by about 50%, and this amount was then used in the next damage experiment. Figure 6C displays the pattern of cell viability after the MLg cells were pre-incubated with BLM for 24 h and then cultured for another 24 h in the different micellar systems. Compared with the BLM model group, all of the groups treated with free IPT, IPT@TFS micelles and IPT@NAC-TFS micelles showed different levels of cell death. The IPT@NAC-TFS micelles showed an increase in the number of viable cells in all cohorts. It is because of this that IPT can protect the MLg cells and also increase the cytoprotective effect by micellar wrapping.

#### 3.9.2. Detection of Intracellular ROS Levels by Flow Cytometry

BLM causes an increase in the ROS in MLg cells. Persistent oxidative stress is well known to be one of the reasons for the development of pulmonary fibrosis [57]. Flow cytometry was used to assess the levels of ROS inside the cells in all the experimental groups (Figure 6D,E). A large number of ROS were present in the BLM group, and all therapies reduced the increase in ROS to some extent. Micelle treatment reduced the amount of intracellular ROS in the free IPT control group, and among all the mixed micelles, IPT@NAC-TFS micelles had the best antioxidant effect in terms of ROS scavenging (*p* < 0.01). IPT has an inherent ROS-regulating effect on the ROS of the cell, and covalently linked NAC can also serve as an antioxidant; thus, it has been found that IPT@NAC-TFS micelles can reduce intracellular ROS.

#### 3.9.3. Oxidative Stress Damage

Too many ROS can cause oxidative stress, reduce the amount of SOD in cells, promote lipid peroxidation, and, thus, increase MDA levels [58,59,60]. Therefore, a high amount of oxidative damage can be considered to impair the activity of SOD and raise MDA levels [61]. As shown in Figure 6F,G, the BLM group had higher MDA levels and lower SOD activity than the uninjured control group; thus, it was determined that an in vitro cellular oxidative injury system had been built. All drug-treated groups exhibited decreased MDA and increased SOD (*p* < 0.01), and this was different from the BLM model group. Therefore, the above preparation can reduce cell damage caused by BLM and improve the antioxidant defense ability of cells. Among all the test materials, IPT@NAC-TFS micelles had a good effect on antioxidant protection and the reduction of lipid peroxidation. A group of NAC at the surface can reduce free radicals and promote the repair of oxidation. In addition, it can increase the quantity of IPT inside the cell. Meanwhile, improved cellular uptake enables micelles to deliver abundant IPT intracellularly, jointly contributing to the protective effects in MLg cells.

### 3.10. Pharmacokinetic Study

**Figure 7:**
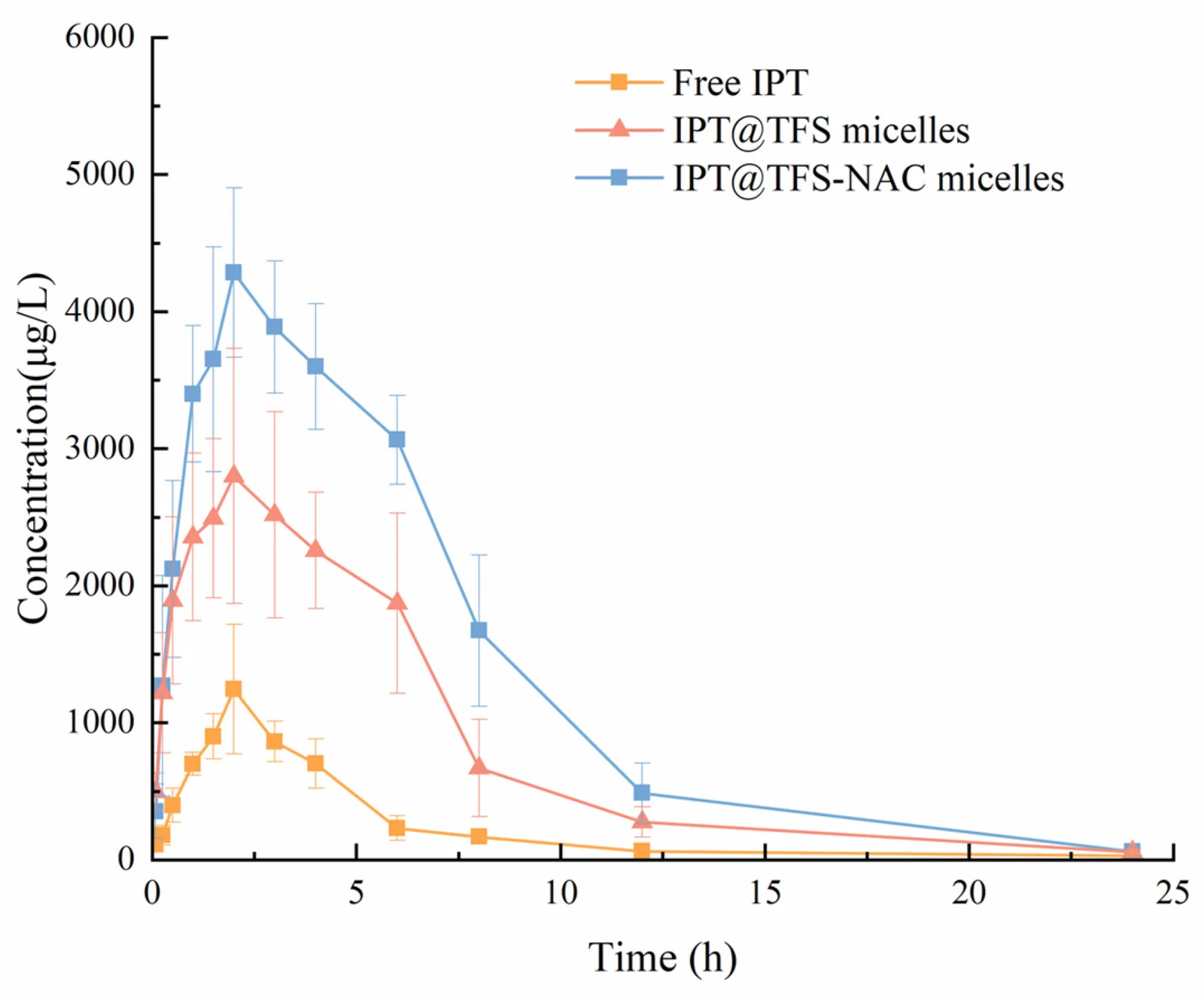
Mean plasma concentration–time profiles of IPT in rats following oral administration of free IPT, IPT@TFS micelles and IPT@NAC-TFS micelles. (mean ± SD, *n* = 5).

The standard curve for IPT was A_IPT_/A_IS_ = 0.4471C − 0.0025 (R = 0.9997). This method exhibited good linearity within the range of 43.50–17,400.00 μg/L. The plasma concentration–time curves of free IPT, IPT@TFS micelles, and IPT@TFS-NAC micelles administered orally at 100 mg/kg to rats are shown in Figure 7 (mean ± SD, *n* = 5). Pharmacokinetic parameters are listed in Table 2 (mean ± SD, *n* = 5). Compared with the free IPT group, both the IPT@TFS micelles and IPT@TFS-NAC micelles groups exhibited significantly increased IPT C_max_ and AUC_0–t_ (*p* < 0.01). The AUC_0–t_ values of IPT in the IPT@TFS micelles and IPT@TFS-NAC micelles groups were equal to 368.87% and 606.76% of the value observed in the free IPT group, respectively. This indicates that both micelles effectively promote the oral absorption of IPT and enhance its relative bioavailability. Compared to the IPT@TFS micelles group, the IPT@TFS-NAC micelles group exhibited significantly higher C_max_ and AUC_0–t_ values for IPT (*p* < 0.05). The improved oral absorption after NAC functionalization can be reasonably explained by the strengthened mucus penetration and enhanced transepithelial transport capacity of IPT@NAC-TFS micelles, consistent with the above in vitro cellular experimental results.

### 3.11. Intestinal Enrichment Experiment

**Figure 8:**
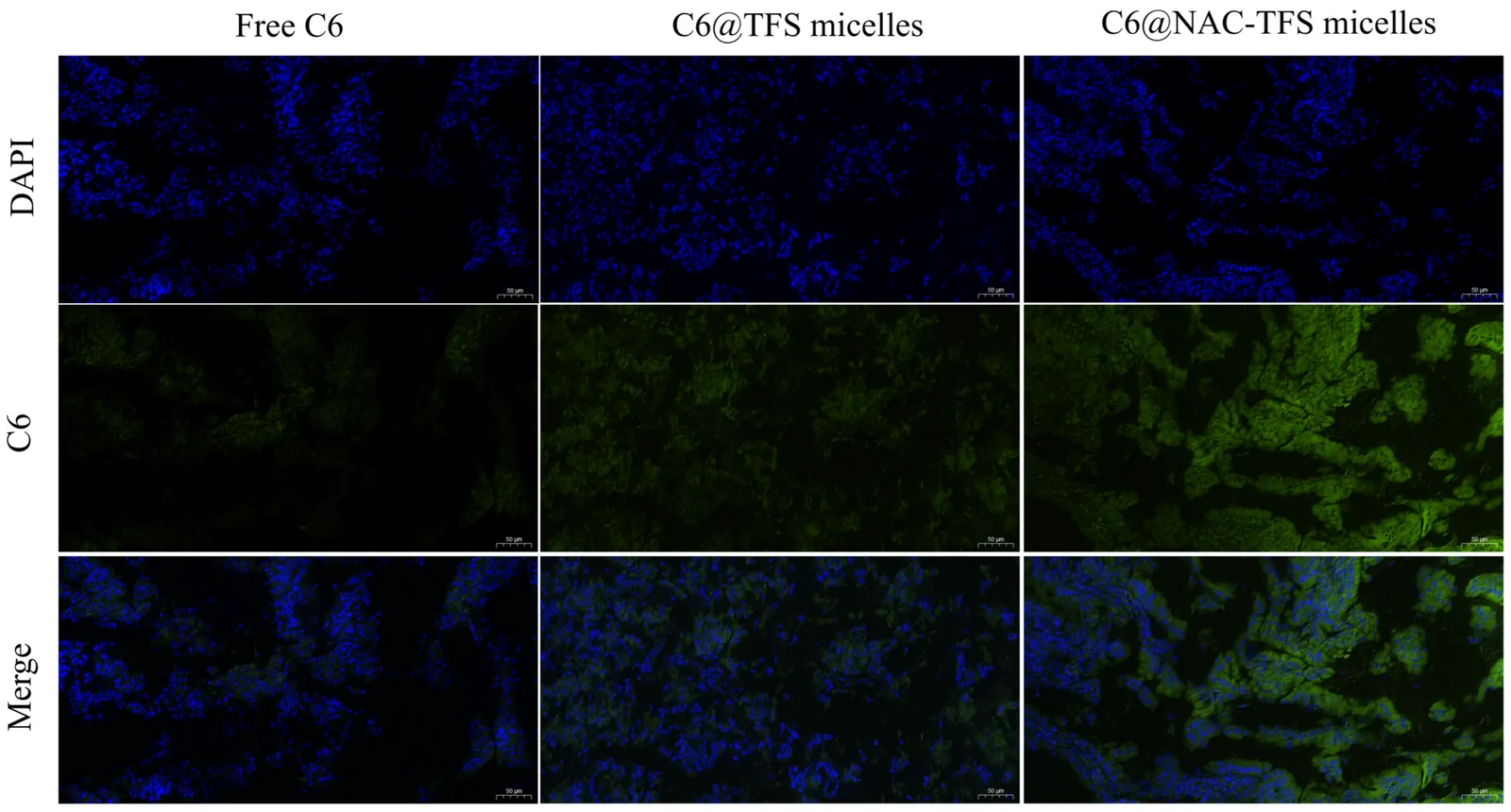
CLSM observation of intestinal tissue sections after oral administration of free C6, C6@TFS micelles and C6@NAC-TFS micelles in rats.

Confocal laser scanning microscopy revealed faint, sporadic fluorescence signals in intestinal tissues for the free C6 group (Figure 8). The C6@TFS micelles group generated stronger fluorescence, predominantly distributed within intestinal epithelial layers. In comparison, intestinal sections treated with C6@NAC-TFS micelles exhibited the most robust fluorescence, with widespread, dense signals covering the whole intestinal mucosa and epithelial cells. Such enhanced intestinal accumulation can be attributed to NAC surface modification. Immobilized NAC moieties enable micelles to penetrate the mucus barrier more efficiently, extend mucosal residence time, and promote subsequent uptake by epithelial cells, thus increasing local drug concentrations. Taken together, these results confirm that NAC-functionalized mixed micelles facilitate drug accumulation in intestinal tissues and achieve efficient intestinal-targeted delivery.

## 4. Conclusions

This study successfully synthesized a dual-functional TPGS-NAC conjugate and fabricated stable, uniformly dispersed IPT@NAC-TFS hybrid micelles with Pluronic^®^ F108 and Lipoid^®^ S-100. Covalent NAC modification improved the thermodynamic stability of micelles and endowed them with excellent mucus-lytic capability, effectively breaking the intestinal mucus barrier, promoting mucosal penetration, and increasing intestinal drug enrichment. The TPGS component simultaneously enhanced epithelial permeability and reduced cellular drug efflux. It transformed the transmembrane transport mode of IPT from passive diffusion to active transport, thereby significantly enhancing the intestinal absorption efficiency. Moreover, IPT@NAC-TFS micelles exhibited prominent antioxidant protective effects in BLM-injured MLg fibroblasts by scavenging intracellular ROS, restoring SOD activity and reducing MDA accumulation. In vivo pharmacokinetic results further verified that NAC functionalization significantly improved the oral bioavailability of IPT. Overall, this multifunctional micellar system effectively solves the key defects of oral IPT delivery, including poor water solubility, multi-barrier absorption limitations and oxidative cell damage. It provides a promising mixed micelle strategy for the oral formulation development of poorly soluble natural antifibrotic agents.

## Figures and Tables

**Table 1:** The apparent permeability coefficient and efflux ratio of IPT (mean ± SD, *n* = 3).

Sample	P_app_ × 10^−5^ (cm/s)		Efflux Ratio P_app_ (BL→AP)/P_app_ (AP→BL)
	AP→BL	BL→AP	
Free IPT	0.713 ± 0.059	6.271 ± 0.021	0.881
IPT@TFS micelles	1.185 ± 0.037 **	5.415 ± 0.129 **	0.457
IPT@NAC-TFS micelles	1.224 ± 0.091 **^##^	4.324 ± 0.172 **^##^	0.353

** *p* < 0.01 vs. free IPT. ^##^ *p* < 0.01 vs. IPT@TFS micelles.

**Table 2:** Pharmacokinetic Parameters of IPT. (mean ± SD, *n* = 5.)

Parameters	Free IPT	IPT@TFS Micelles	IPT@NAC-TFS Micelles
AUC_0–t_ (μg/L/h)	5361.55 ± 866.38	19,777.01 ± 4763.46 **	32,531.90 ± 4622.08 **^##^
AUC_0–∞_ (μg/L/h)	5527.06 ± 945.47	19,904.07 ± 4862.99 **	32,786.68 ± 4843.75 **^##^
t_1/2_ (h)	5.04 ± 2.25	3.00 ± 0.69	3.07 ± 0.57
T_max_ (h)	2.20 ± 0.45	2.30 ± 0.98	1.90 ± 0.22
C_max_ (μg/L)	1051.34 ± 144.16	2885.33 ± 898.49 **	4499.61 ± 399.86 **^##^

** *p* < 0.01 vs. free IPT. ^##^ *p* < 0.01 vs. IPT@TFS micelles.

## Data Availability

The original contributions presented in this study are included in the article. Further inquiries can be directed to the corresponding authors.

## Abbreviations

The following abbreviations are used in this manuscript:

^1^H-NMR	proton nuclear magnetic resonance
BLM	bleomycin
C6	Coumarin 6
CDI	N,N’-carbonyldiimidazole
CMC	critical micelle concentration
CMC-Na	sodium carboxymethyl cellulose
DL	drug loading
DMSO	dimethyl sulfoxide
DMSO-d_6_	deuterated dimethyl sulfoxide
EDC·HCl	1-Ethyl-(3-dimethylaminopropyl) carbodiimide Hydrochloride
EE	encapsulation efficiency
FTIR	Fourier-transform infrared spectroscopy
HCl	hydrochloric acid
HOBt	1-hydroxybenzotriazole
IC_50_	half-maximal inhibitory concentration
IPT	Imperatorin
IS	internal standard
KBr	potassium bromide
MDA	malondialdehyde
MLg	mouse lung fibroblast cell line
NAC	N-acetylcysteine
P_app_	apparent permeability coefficient
PDI	polydispersity index
PEG 20000	polyethylene glycol
PEO	polyethylene oxide
P-gp	P-glycoprotein
ROS	reactive oxygen species
SD rats	Sprague–Dawley rats
SOD	superoxide dismutase
TEM	transmission electron microscopy
TPGS	D-α-Tocopherol polyethylene glycol 1000 succinate
TFS	TPGS/Pluronic^®^ F108/Lipoid^®^ S-100
UPLC	ultra-high-performance liquid chromatography
X-RD	X-ray diffraction
